# *Haem Oxygenase 1* is a potential target for creating etiolated/albino tea plants (*Camellia sinensis*) with high theanine accumulation

**DOI:** 10.1093/hr/uhac269

**Published:** 2022-12-02

**Authors:** Ziping Chen, Shijia Lin, Tingting Chen, Mengxue Han, Tianyuan Yang, Yan Wang, Shilai Bao, Zhougao Shen, Xiaochun Wan, Zhaoliang Zhang

**Affiliations:** State Key Laboratory of Tea Plant Biology and Utilization, Anhui Agricultural University, Hefei, 230036, China; Anhui Promotion Center for Technology Achievements Transfer, Anhui Academy of Science and Technology, Hefei, 230031, China; State Key Laboratory of Tea Plant Biology and Utilization, Anhui Agricultural University, Hefei, 230036, China; State Key Laboratory of Tea Plant Biology and Utilization, Anhui Agricultural University, Hefei, 230036, China; State Key Laboratory of Tea Plant Biology and Utilization, Anhui Agricultural University, Hefei, 230036, China; State Key Laboratory of Tea Plant Biology and Utilization, Anhui Agricultural University, Hefei, 230036, China; State Key Laboratory of Tea Plant Biology and Utilization, Anhui Agricultural University, Hefei, 230036, China; State Key Laboratory of Molecular Developmental Biology, Institute of Genetics and Developmental Biology, Chinese Academy of Sciences, Beijing, 100101, China; School of Life Sciences, University of Chinese Academy of Sciences, Beijing, 100049, China; State Key Laboratory of Tea Plant Biology and Utilization, Anhui Agricultural University, Hefei, 230036, China; State Key Laboratory of Tea Plant Biology and Utilization, Anhui Agricultural University, Hefei, 230036, China; State Key Laboratory of Tea Plant Biology and Utilization, Anhui Agricultural University, Hefei, 230036, China

## Abstract

Theanine content is highly correlated with sensory quality and health benefits of tea infusion. The tender shoots of etiolated and albino tea plants contain higher theanine than the normal green tea plants and are valuable materials for high quality green tea processing. However, why these etiolated or albino tea plants can highly accumulate theanine is largely unknown. In this study, we observed an *Arabidopsis* etiolated mutant *hy1–100* (mutation in *Haem Oxygenase 1*, *HO1*) that accumulated higher levels of glutamine (an analog of theanine). We therefore identified CsHO1 in tea plants and found CsHO1 is conserved in amino acid sequences and subcellular localization with its homologs in other plants. Importantly, *CsHO1* expression in the new shoots was much lower in an etiolated tea plants ‘Huangkui’ and an albino tea plant ‘Huangshan Baicha’ than that in normal green tea plants. The expression levels of *CsHO1* were negatively correlated with theanine contents in these green, etiolated and albino shoots. Moreover, *CsHO1* expression levels in various organs and different time points were also negatively correlated with theanine accumulation. The *hy1–100* was hypersensitive to high levels of theanine and accumulated more theanine under theanine feeding, and these phenotypes were rescued by the expression of *CsHO1* in this mutant. Transient knockdown *CsHO1* expression in the new shoots of tea plant using antisense oligonucleotides (asODN) increased theanine accumulation. Collectively, these results demonstrated CsHO1 negatively regulates theanine accumulation in tea plants, and that low expression *CsHO1* likely contributes to the theanine accumulation in etiolated/albino tea plants.

## Introduction

Tea plant (*Camellia sinensis*) is one of most broadly cultured economic crops globally. It contains abundant secondary metabolites that account for its sensory quality and health benefits. Among these, theanine (γ-glutamylethylamide), a unique non-proteinogenic amino acid in tea plants, is the component conferring the ‘umami’ taste of green tea infusion [1]. In addition, theanine has many health benefits including promoting relaxation and calming, improving cognitive function (learning and memory), and promoting sleep for anxiety insomnia, in human and animals [2, 3]. It is speculated that theanine increases the levels of inhibitory neurotransmitters, including γ-aminobutyric acid (GABA), serotonin and dopamine [4, 5]. Therefore, theanine content largely determines the quality and price of green teas [6].

Theanine is mainly synthesized in the root of tea plants from glutamate (Glu) and ethylamine (EA) by theanine synthetase (CsTSI) [7, 8]. Glutamine synthetases (GS) can also catalyze theanine synthesis from Glu and EA. It is proposed that CsGS1.1 and CsGS2 also contribute to theanine synthesis in shoot tissues [9]. Glutamate synthases (GOGATs) and glutamate dehydrogenases (GDHs) catalyze Glu biosynthesis in tea plants, and alanine decarboxylase (CsAlaDC) synthesizes EA from alanine [10, 11]. The genesencoding GOGATs, GDHs and CsAlaDC were identified recently [8, 11, 12]. Theanine catabolism is probably catalyzed by CsPDX2.1in tea plants [13]. These processes of theanine biosynthesis andcatabolism are affected by many environmental factors, especially nutrient levels, light intensity, and salinity, etc. [14–16].

Albino and etiolated tea plant cultivars, such as ‘HuangshanBaicha’ (HSBC) and ‘Huangkui’ (HK), produce white or yellowshoots and are of great value for high quality green tea productionand are highly desired by the tea market. That is because the teas produced from the white or white tender shoots contains much higher theanine and lower polyphenols, and therefore have an improved ‘umami’ taste and weak bitterness and stringency[17]. It was proposed that mutations in genes encoding proteins in photosynthetic pigment biosynthesis and degradation,the chloroplastic DOXP/MEP pathway, and chloroplast-nucleus signaling, are responsible for the albino phenotype of tea plants[18–20]. However, why theanine highly accumulates in albino andetiolated tea plants is still largely unknown.

Haem oxygenases (HOs) catalyze haem degradation into carbon monoxide (CO), free iron (Fe^2+^), and biliverdin-IXα (BV-IXα) [21]. In *Arabidopsis*, HOs include four members: HO1, HO2, HO3, and HO4. Within these members, HO1 is the most highly expressed and is the main enzymatic HO form [22]. The mutation of HO1 significantly decreased chlorophyll accumulation, which was possibly caused by the high accumulation of haem, which feed back inhibited chlorophyll synthesis [22–24]. In soybean plants, HO1 was proposed to protect nitrogen metabolism in nodules against salt stress [25]. In rice, *HO1* expression and HO1 activity were shown to be induced by ammonium and involved in protecting against high ammonium-induced leaf chlorosis [26]. These studies led us to ask whether HO1 is involved in the regulation of chlorophyll biosynthesis and nitrogen metabolism in tea plants.

The etiolated or albino appearance is also favored in many other horticulture plants, including vegetables, fruits, and tea plants. Amino acids are also important quality components of these horticultural products. It is important to dissect the relation between etiolated/albino appearance and amino acid accumulation, and further reveal the underlying regulatory mechanism. The knowledge will be critical for creating etiolated/albino cultivars with higher amino acid contents. In this study, we found an *Arabidopsis* etiolated mutant *hy1–100*, the mutant of *HO1*, hyper-accumulated glutamine (Gln), the analog of theanine. The following correlation analyses demonstrated that *CsHO1* expression significantly and negatively correlated with theanine accumulation in tea plants. In the theanine feeding experiment, *hy1–100* mutant was found to hyper-accumulate theanine, and the expression *CsHO1* in the *hy1–100* mutant complemented the phenotype. Finally, transient knockdown *CsHO1* expression using antisense oligonucleotide (asODN) significantly increased theanine accumulation in new shoots of tea plants. These results suggested CsHO1 negatively modulates theanine accumulation. This study provided insights into why albino and etiolated tea plants accumulate high levels of theanine and is of potential importance for the development of novel albino and etiolated cultivars of tea plants.

## Results

### Hyper-accumulation of Gln or theanine in etiolated *Arabidopsis hy1–100* mutant and tea plant cultivar ‘Huangkui’

**Figure 1:**
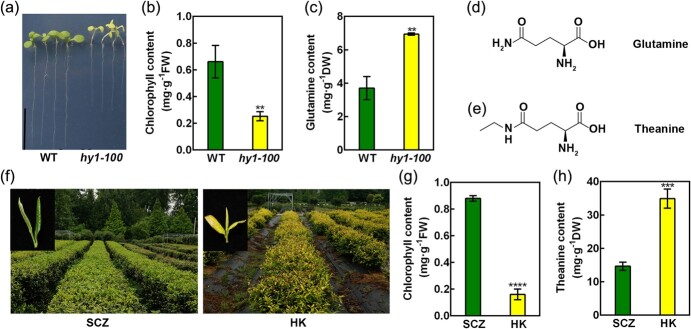
Etiolated *Arabidopsis* and tea plant seedlings accumulated higher level of glutamine or theanine. **A**–**C** The phenotype (**A**), chlorophyll contents (**B**), and glutamine contents (**C**) in *Arabidopsis* wild type (WT) and *hy1–100* mutant. **D**–**E** The formula of glutamine (**D**) and theanine (**E**). **F**–**H** The leaf color (**F**), chlorophyll contents (**G**) and theanine contents (**H**) in a green tea plant cultivar ‘Shuchazao’ (SCZ) and an etiolated cultivar ‘Huangkui’ (HK). Data represent mean ± SE (*n* = 3). Asterisks indicate significant differences from SCZ or WT based on Student’s *t*-test (^**^P < 0.01, ^***^P < 0.001, ^****^P < 0.0001).

Previous studies revealed that Haem Oxygenase 1 (HO1/HY1) plays an essential role in chlorophyll synthesis in *Arabidopsis* [27]. Here, we verified that the *Arabidopsis HO1* mutant, *hy1–100*, showed etiolated phenotype and contained much lower chlorophyll (Fig. 1A and B). Interestingly, we found *hy1–100* accumulated more Gln, the analog theanine, than wild-type (WT) seedlings (Fig. 1C–E). Similarly, an etiolated tea plant ‘Huangkui’ (HK), accumulated a lower level of chlorophyll and a higher level of theanine than a normal green tea plant cultivar ‘Shuchazao’ (SCZ) (Fig. 1F–H). These results suggested HO1 regulates Gln accumulation in *Arabidopsis*, and led us to study whether HO1 homolog in tea plants regulates theanine accumulation.

We next examined the accumulation of some other amino acids in WT and *hy1–100*. The results showed that *hy1–100* also accumulated more glutamate, aspartate, and proline, and accumulated less glycine, serine, cysteine, and ornithine, than the WT (Fig. S2, see online supplementary material). The results indicated that the mutation of *AtHO1* affects the accumulation of many amino acids.

### CsHO1 is conserved in sequence and subcellular localization

**Figure 2:**
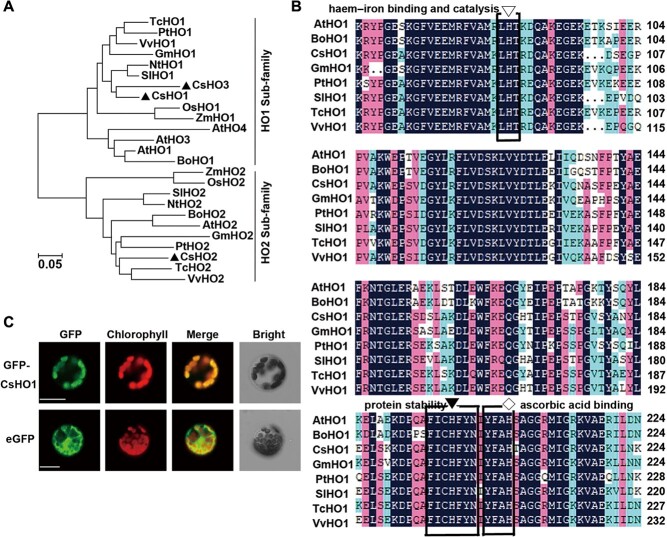
Phylogenetic tree, multiple sequence alignment, and sub-cellular localization of CsHO1. **A** Phylogenetic relationship of HOs in *Camellia sinensis* (Cs), *Arabidopsis thaliana* (At), *Brassica oleracea* (Bo), *Nicotiana tabacum* (Nt), *Solanum lycopersicum* (Sl), *Vitis vinifera* (Vv), *Populus tremula* (Pt), *Theobroma cacao* (Tc), *Glycine max* (Gm), *Oryza sativa* (Os), and *Zea mays* (Zm). The scale bar corresponds to 0.05 estimated amino acid substitutions per site. **B** Alignment of CsHO1 with other plant HO1 proteins. Identical (100%), conservative (75–99%), and blocks (50–74%) of similar amino acid residues are shaded in deep blue, cherry red, and light blue, respectively. The conserved histidine residues involved in heme-iron binding and catalysis, protein stability, and ascorbic acid binding were marked by a white reverse triangle, a black reverse triangle and a white diamond, respectively. **C** Subcellular localization of CsHO1 protein in *Arabidopsis* protoplasts. The photographs were taken in the green channel, in the red channel, in their combination, or in the bright channel. Scale bars represent 20 μm.

Subsequently, we searched HO homologous in tea plants through the Tea Plant Information Archive (TPIA) (http://tpdb.shengxin.ren/) [28], and identified three putative CsHOs. The phylogenetic tree showed that CsHOs belonged to two sub-families: HO1 sub-family and HO2 sub-family. CsHO1 and CsHO3 clearly grouped into HO1 sub-family with HO1, HO3, and HO4 in *Nicotiana tabacum*, *Solanum lycopersicum*, *Arabidopsis thaliana*, *Brassica oleracea*, *Vitis vinifera*, *Populus tremula*, *Theobroma cacao*, *Glycine max*, *Oryza sativa*, and *Zea mays* (Fig. 2A). Gene expression data from the TPIA also showed that, within three *CsHOs*, *CsHO1* was the most highly expressed in different tissues (Fig. S1, see online supplementary material). Several highly conserved motifs of HO1s in various plant species were identified (Fig. 2B), indicating that CsHO1 is conserved with HO1s in other plants.

To assess the subcellular localization of CsHO1, we fused CsHO1 to green fluorescent protein (GFP) driven by the CaMV 35S promoter (35S::*GFP-CsHO1*). The 35S::*GFP-CsHO1* and the control 35S::*GFP* were transformed into *Arabidopsis* protoplasts. As shown in Fig. 2C, the free GFP fluorescence was visualized in both cytosol and nucleus. In contrast, the fluorescent signal of GFP-CsHO1 overlapped with that of the auto-fluorescence of chlorophylls in chloroplast. These results indicated that CsHO1 localizes in the chloroplast. This localization is consistent with HO1 in *Medicago sativa*, *Lycopersicon esculentum*, *Triticum aestivum*, and *Cucumis sativus* [29–32].

### *CsHO1* expression was much lower in the new shoots of etiolated and albino tea plants

**Figure 3:**
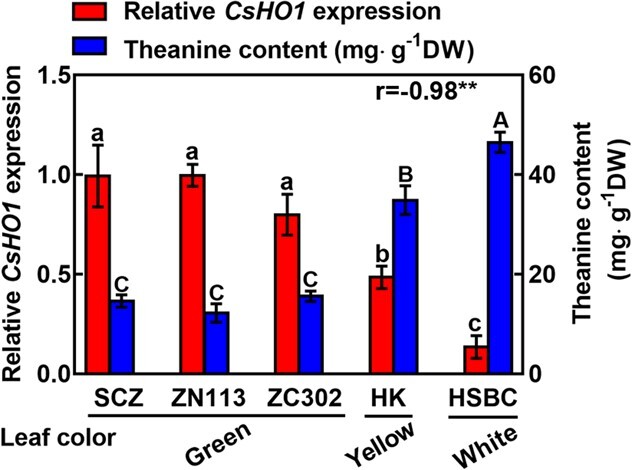
*CsHO1* expression and theanine contents in normal, etiolated, and albino tea plants. *CsHO1* expression and theanine contents in the buds of tea cultivars SCZ, ZN113, ZC302, HK, and HSBC. *CsHO1* relative expression was analysed using real-time PCR. Relative *CsHO1* expression level in the bud of SCZ was set as 1. Correlation coefficient between *CsHO1* expression of theanine contents was shown as r. Data is means ± SE of three biological replicates. The correlation coefficient between theanine content and *CsHO1* expression was analysed via Pearson correlation (^**^P < 0.01).

To access the role of CsHO1 in modulating chlorophyll content and theanine accumulation, we compared the expression of *CsHO1* in the new shoots of three normal green tea plant cultivars (SCZ, Zhenong 113[ZN113], Zhongcha 302[ZC302]), one etiolated cultivar HK and one albino cultivar HSBC. The results showed that the expression levels in HK and HSBC were much lower than those in normal green cultivars (Fig. 3). In this and previous studies, the chlorophyll contents in HK and HSBC were shown to be significantly lower than green cultivars [33, 34] (Fig. 1G). Therefore, this result implied a positive role of CsHO1 in chlorophyll biosynthesis in tea plants just like its homologs in other plants [23, 24, 27, 35].

Next, we measured the theanine contents in the new shoots of these cultivars. The theanine contents showed an opposite pattern to that of *CsHO1* expression in these cultivars, with higher theanine contents in HK and HSBC (Fig. 3). The correlation coefficient between *CsHO1* expression and theanine contents was further analysed. The result indicated that *CsHO1* expression was highly and negatively correlated with theanine contents (r = −0.98, *P* < 0.001). The results supported the notion that CsHO1 negatively regulates theanine accumulation in tea plants.

### *CsHO1* expression negatively correlated with theanine contents in spatiotemporal levels in tea plants

**Figure 4:**
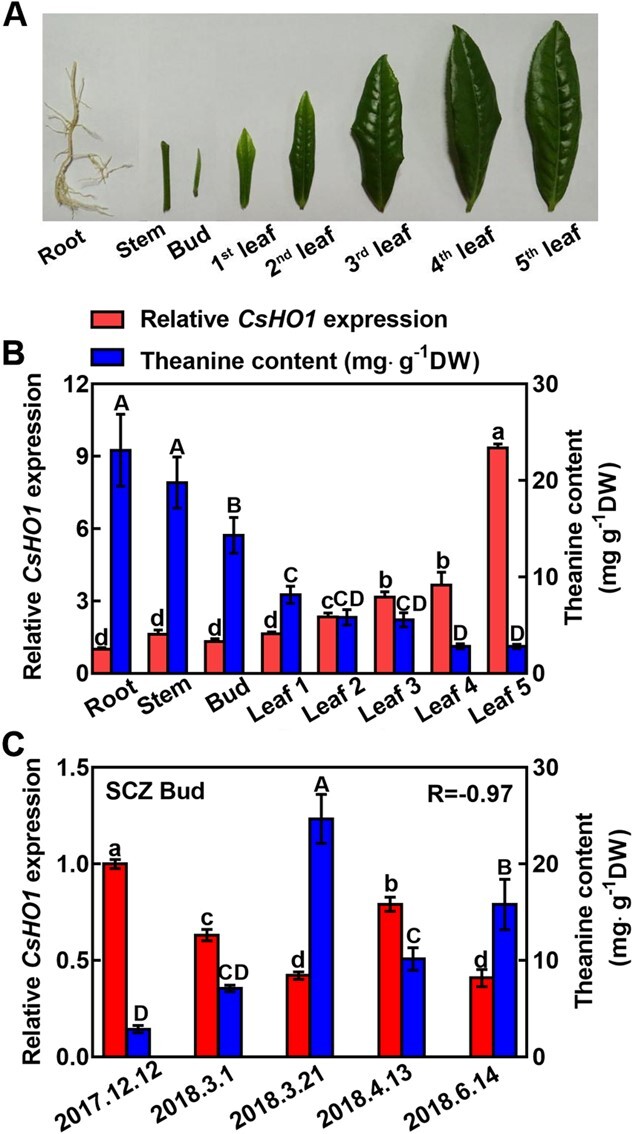
The negative correlation between *CsHO1* expression and theanine content in various organs and at different time points. **A**–**B** Tissue samples (**A**), *CsHO1* expression and theanine contents in these organs of SCZ. **C**  *CsHO1* expression and theanine contents in the buds of SCZ at different time points including 12 December (Dec 12), 1 March (Mar 1), 23 March (Mar 23), 13 April (Apr 13), and 14 June (Jun 14). These samples and theanine contents at Dec 12, Mar 1, Mar 23, and Apr 13 were described by Dong *et al*. (2020) [36]. *CsGADPH* was used as an internal control. In (**B**), relative *CsHO1* expression level in the root was set as 1. In (**C**), relative *CsHO1* expression level in the bud at Dec 12 was set as 1. Correlation coefficient between *CsHO1* expression of theanine contents was shown as r. Data are means ± SE of three biological replicates. The correlation coefficient was analysed via Pearson correlation (^*^P < 0.05, ^**^P < 0.01).

To further reveal the role of CsHO1 in theanine accumulation, *CsHO1* expression and theanine accumulation levels in different organs of tea plants were examined. The organs examined are shown in Fig. 4A. In these organs, *CsHO1* expression level also showed an opposite tendency to that of theanine content (Fig. 4B). For example, in the organs including bud, 1st leaf, 2nd leaf, 3rd leaf, 4th leaf, and 5th leaf, the expression level of *CsHO1* showed a pattern of gradual increase; in contrast, theanine contents showed a pattern of gradual decrease. Further correlation analysis indicated that *CsHO1* expression was significantly and negatively correlated with theanine accumulation in these organs.

Theanine accumulation in the new shoots of tea plants is highly dynamic at different time points. Therefore, we next examined the *CsHO1* transcript levels and theanine contents in the leaf buds of tea plant at five time points, including 12 December, 1 March, 23 March, 13 April, and 14 June. Again, *CsHO1* expression showed an opposite pattern to that of theanine contents, with a correlation coefficient − 0.97 (Fig. 4C). These results further implied that CsHO1 negatively modulates theanine accumulation in tea plants.

### Both AtHO1 and CsHO1 negatively regulated theanine accumulation in *Arabidopsis* fed with theanine

**Figure 5:**
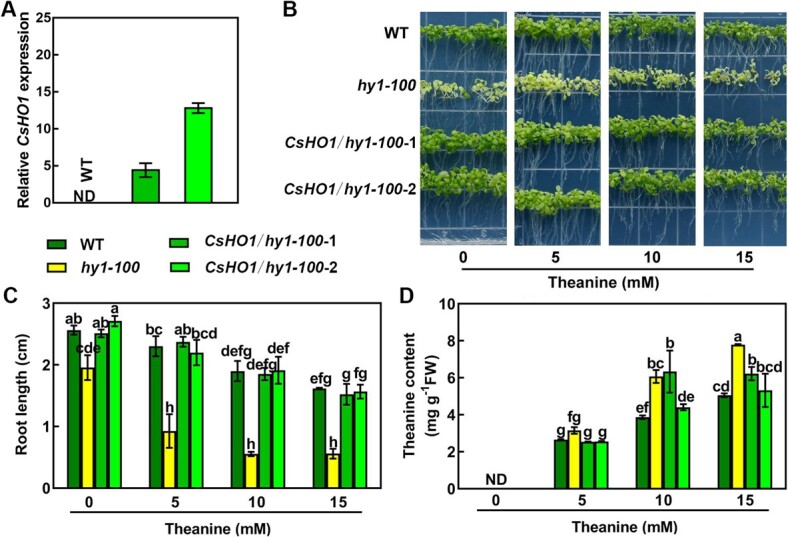
Theanine contents in *hy1–100* and *CsHO1* transgenic *hy1–100* lines (*CsHO1/hy1–100*-1*, CsHO1/hy1–100*-2). (**A**) *CsHO1* expression in two *CsHO1* transgenic *hy1–100* lines (*CsHO1/hy1–100*-1*, CsHO1/hy1–100*-2). The expression levels were relative to that of *AtACTIN7*. Wild-type (WT) was used a negative control. ND, not detected. (**B**) The growth phenotype of WT, *hy1–100*, and two *CsHO1* transgenic lines (*CsHO1/hy1–100*-1*, CsHO1/hy1–100*-2) in MS medium supplemented with 0, 5, 10, or 15 mM theanine for 14 d. Root length (**C**) and theanine contents (**D**) were then determined in these *Arabidopsis* lines. Data are mean ± SE of three biological replicates. Letters denote significant difference at *P* < 0.05 according to Tukey’s multiple comparisons.

Previously studies observed that yeast, *Arabidopsis*, and tomato can also uptake and degrade theanine [36, 37]. Therefore, we next tested whether AtHO1 regulates theanine accumulation in *Arabidopsis* fed with theanine. Various concentrations of theanine (0, 5, 10, and 15 mM) were added into the MS medium to feed *Arabidopsis* wild type (WT) and *hy1–100* mutant. The *hy1–100* mutant showed hypersensitivity to high concentrations of theanine (10 and 15 mM) in terms of the inhibiting effects of theanine in root growth (Fig. 5B and C). At the same condition, theanine accumulation in *hy1–100* mutant was also significantly higher than in the WT (Fig. 5D). These results indicated that AtHO1 regulated theanine accumulation in *Arabidopsis* fed with theanine.

To verify the role of *CsHO1* in theanine accumulation, 35S promoter-driven *CsHO1* was transferred into *hy1–100* mutant. The expression of *CsHO1* in *hy1–100* mutant rescued the etiolated phenotype of *hy1–100* in two independent transgenic lines (*CsHO1/hy1–100*-1*, CsHO1/hy1–100*-2) (Fig. 5A and B). This result suggested that CsHO1 is functionally conserved with AtHO1 in regulating chlorophyll biosynthesis. Furthermore, *CsHO1* expression also rescued the glutamate hyper-accumulation, and partially rescued the glutamine hyper-accumulation, in *hy1–100* mutant (Fig. S3, see online supplementary material). Meanwhile, under 10 and 15 mM feeding conditions, the hyper-sensitivity to theanine and theanine hyper-accumulation phenotypes of *hy1–100* mutant were also rescued by *CsHO1* expression (Fig. 5B–D). These results provided further genetic evidence for the regulatory roles of CsHO1 in chlorophyll biosynthesis and theanine accumulation.

### Knockdown *CsHO1* expression increased theanine accumulation in the new shoots

**Figure 6:**
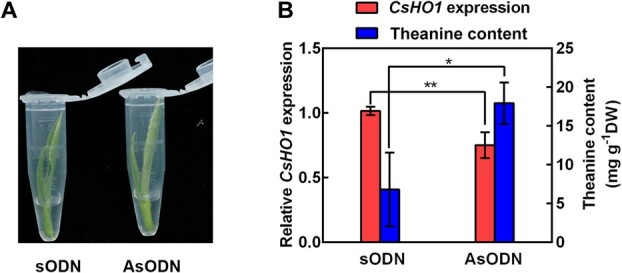
Theanine accumulation in the new shoots with suppressed *CsHO1* expression. (**A**) The new shoots were subjected to 40 μM sDON or asODN for 24 h. (**B**) *CsHO1* expression was performed and theanine contents in these shoots. Data are mean ± SE of three biological replicates. Asterisks indicate significant differences from sODN treatment based on Student’s *t*-test (^*^P < 0.05, ^**^P < 0.01).

Antisense oligonucleatides are broadly used to transiently inhibit gene expression in tea plants [38, 39]. To provide more evidence for the negative role of CsHO1 in theanine accumulation in tea plants, we treated the new shoots of SCZ with the *CsHO1*-specific sense oligonucleatides (sODN) or antisense oligonucleatides (asODN) for 24 h (Fig. 6A). The expression level of *CsHO1* was significantly repressed by asODN treatment compared with the control sODN treatment (Fig. 6B). Meanwhile, theanine accumulation was significantly increased in the asODN-treated new shoots (Fig. 6B). These results further indicated CsHO1 regulates theanine accumulation in tea plants.

## Discussion

### HO1 links the etiolated or albino appearance with amino acid accumulation in plant

Along with haem degradation, HO1 performs the process of phytochrome chromophore synthesis, and thus is essential for light signaling [21]. In addition, HO1 also regulates chlorophyll biosynthesis, stomatal regulation, salt and drought tolerance, and rootdevelopment [22]. However, its role in amino acid accumulation was not investigated yet. In this study, we observed the accumulation of glutamate, aspartate and proline was higher, and the accumulation of glycine, serine, cysteine, and ornithine was lower, in *hy1–100*, an etiolated *Arabidopsis* mutant. These results, for the first time, linked HO1 amino acid accumulation with etiolated phenotype in plant.

A large number of studies reported that etiolated/albino tea plants accumulated higher levels of amino acids [17, 40–44]. Higher accumulation of glutamate, glutamine, arginine, asparagines was also reported in other *Arabidopsis* albino or pale-green mutants [45]. Given that both etiolated/albino appearanceand amino acid contents are important for many horticultural plants, it is important to further study the association between these two traits and the underlying regulatory roles of HO1 in other horticultural plants.

### CsHO1 is likely involved in the dynamic regulation of theanine accumulation in new shoots of tea plants

Since theanine was discovered, its accumulation has been widely studied [7, 46]. The accumulation of theanine was dynamically regulated by seasons, light, and nitrogen supplements [14, 15, 47]. Our previous studies indicated that theanine content in leaf bud was low in winter, and underwent a significant increase in early spring, and then decreased in the middle of April [47], suggesting that L-theanine accumulation is dynamic and highly regulated. Our recent study suggested that glutamate dehydrogenase 2.1 (CsGDH2.1)-catalyzed glutamate catabolism negatively regulates theanine accumulation in the late-spring new shoots of tea plants [48].

In this study, we revealed that CsHO1, the homolog of AtHO1, also negatively regulates theanine accumulation. *CsHO1* expression level decreases in early spring and increases in late spring, being opposite to the changing pattern of theanine accumulation in the new shoots of plants (Fig. 4C), suggested that CsHO1 is likely also involved in the regulation of theanine accumulation in spring, especially in late spring.

Given that the rapid decrease of theanine level in the late spring leads to the decline of processed green tea quality, it will be intriguing to develop a cultural practice to inhibit *CsHO1* expression and further to improve the theanine content in the late spring. It was reported that UV-B radiation induced the expression of *HO1* in soybean, and this induction was arrested by pretreatment of ascorbic acid former to UV-B treatment [49]. It is worth a try to test the effects of ascorbic acid treatment on *CsHO1* expression and theanine content in late spring.

### Low expression of CsHO1 contributes to theanine accumulation in etiolated or albino tea plants

Lots of studies have showed that etiolated or albino tea leaves accumulate more theanine than the normal green tea leaves [17, 42]. We also usually observed that tea leaves are pale green in early spring and are dark green in the late spring. In contrast, the theanine contents are high in early spring and significantly decrease in late spring [47, 48]. In addition, under shade conditions, tea leaves become dark green and theanine contents decrease in these leaves [15]. These studies and observations implied that theanine accumulation in tea leaves is negatively correlated with chlorophyll contents.

Albino or etiolated tea plant mutants are ideal material for studying the correlation between theanine and chlorophyll contents. In this study, we found *CsHO1* expression in etiolated and albino tea plant cultivars was much lower than in the normal green cultivars (Fig. 3). More importantly, when *CsHO1* was expressed in the *hy1–100* mutant, the etiolated phenotype and chlorophyll content of this mutant was rescued (Fig.5). The higher theanine accumulation in the mutant fed with theanine was also rescued. Transiently knock-down *CsHO1* expression using asODN in tea plant shoots also significantly increased theanine accumulation (Fig. 6). These genetic analyses provided strong evidence to support the role of *CsHO1* in regulating theanine accumulation in tea plants. Therefore, the low expression of *CsHO1* in these etiolated or albino tea plant likely contributes to the low chlorophyll contents and the high theanine contents (Fig. 7).

Cheng *et al.* (2019) pointed out that weak theanine degradation contributes to the high theanine accumulation in etiolated or albino tea plants [50]; however, the underlying regulatory mechanism is unknown. It is known that glutamate is both the precursor of theanine biosynthesis and the product of theanine degradation [7]. Glutamate is also an important signaling molecule in the regulation of development, stress response and metabolism [51, 52]. Recently, Chen *et al.* (2022) proposed that CsGDH2.1-modulated glutamate content in the new shoots of tea plants represses theanine degradation [48]. Consistently, the glutamate accumulation is higher in *hy1–100* mutant and in etiolated or albino new shoots of tea plants. It is likely that the low expression of *HO1* increases glutamate content, which leads to weak theanine degradation in etiolated or albino tea plants.

In summary, this study revealed a novel regulatory role of CsHO1 in theanine accumulation in tea plants, and also provided a target gene for creating etiolated or albino tea plant germplasm, which is highly needed by the tea industry. HO1 is an enzyme with multiple functions in haem catabolism and the biosynthesis of phytochrome chromophore and CO, and also functions as an important defending system against reactive oxygen species [22]. These processes are critical for chloroplast biosynthesis, light signaling, photosynthesis, development, and stress tolerance. In future, we need to further explore how HO1 regulates the accumulation of amino acids, especially glutamate and theanine.

## Materials and methods

### Plant materials and growth conditions

Eight-year-old tea plant cultivars were used in this study. These cultivars, including ‘Shuchazao’ (SCZ), ‘Zhenong 113’ (ZN113), ‘Zhongcha 302’ (ZC302), ‘Huangkui’ (HK), and ‘Huangshan Baicha’ (HSBC) were grown in the Guohe Tea plantation (31°N, 117°E, China). Organs including roots, stem, leaf buds, and the first to the fifth leaf of new shoots were sampled as indicated.

The *Arabidopsis hy1–100* mutant was from Professors Wenbiao Shen and Yanjie Xie at College of Life Sciences, Nanjing Agricultural University, China. The seeds were stratified at 4°C for 2 d and then transferred into the growth chamber. The condition set was 16 h/8 h day/night light period, 150 μmol m^−2^ s^−1^ light intensity, 23°C/18°C, and 70% relative humidity. *Arabidopsis* wild-type (WT), *hy1–100* mutant and transgenic lines grew for 14 d on MS medium supplemented with 0, 5, 10, or 15 mM theanine.

### Measurement of theanine content

Theanine was extracted and measured as previous described by our laboratory [37].

### Determination of chlorophyll content

Chlorophyll of seedlings was extracted using 95% (v/v) ethanol for 24 h in darkness, and then calculated by examining the absorbance at 649 nm and 665 nm.

### Construct of phylogenetic tree and amino acid sequence aliment

MEGA program (ver 4.1) (Auckland, New Zealand) was used to construct the phylogenetic tree of HOs, by neighbor-joining (NJ) method. The parameters pairwise deletion and *P*-distance model were used. Bootstrap test of phylogeny was performed with 1000 replicates. Alignment of HO sequences was performed with DNAMAN 6.0.3.99 (Lynnon Biosoft Bioinformatic Solutions, https://www.lynnon.com/, USA).

### Subcellular localization analysis

*CsHO1*-specific primers (Table S1, see online supplementary material) were used to amplify the gene from the cDNA library of tea plant cultivar ‘Shuchazao’. The PCR fragment without the stop codon was inserted into the pAN580 vector and fused with green fluorescent protein (GFP). The obtained pAN580-*GFP-CsHO1* and the empty vector pAN580-*GFP* were transformed into *Arabidopsis* protoplasts using polyethylene glycol (PEG)-mediated gene transformation [53]. After being incubated in the dark for 16 h at room temperature, the fluorescence was observed using a laser scanning confocal microscope (LSCM, Olympus FluoView™ FV1000, Japan).

### Antisense oligonucleotides-mediated gene supression

AsODN and sODN sequences were designed using SOLIGO software [54] with the sequence of *CsHO1* cDNA as the input. The sequences were listed in Table S1 (see online supplementary material). The new shoots with one bud and the 1st leaf were treated with 0.1 ml 40 μM AsODN (asODN1, asODN2, asODN3, and asODN410; 10 μM for each) or sODN (sODN1, sODN2, sODN3, and sODN410; 10 μM for each) solution. After 24 h incubation, the new shoots were sampled and stored at −80°C.

### Generation of transgenic *Arabidopsis* plants

The CDS of *CsHO1* was amplified with gene-specific primers (see Table S1, see online supplementary material) and cloned into vector pCAMBIA1302 containing the CaMV35S promoter. The recombinant plasmid was transformed into *Agrobacterium tumefaciens* strain GV3101, and then transformed into *Arabidopsis hy1–100* mutant using the floral dip method [55].

### Measurement of free amino acids

Amino acid contents were measured as previously described [18]. Briefly, amino acids were extracted, centrifuged, and filtered for the amino acid content measurement. Amino acids were measured by the High-Speed Amino Acid Analyzer. The amounts of amino acids were calculated according to the calibration curve of amino acid standards.

### Quantitative real-time RT-PCR analysis

Total RNA was extracted using a RNAprep Pure kit (Tiangen, Beijing, China) and TransZol Up Kit (TransGen Biotech, Beijing, China), respectively. cDNAs were synthesized using an oligo(dT) primer. Using gene-specific primers (Table S1, see online supplementary material), real-time quantitative reverse-transcription (RT) PCR was conducted. The expression levels of genes were normalized to *CsGAPDH* and *AtACTIN7* and presented as values relative to corresponding control samples.

## Acknowledgments

This work was supported by the National Key R&D Program of China (2021YFD1601101), grants from the National Natural Science Foundation of China (32072624), and Anhui Provincial Department of Human Resources and Social Security (2021LXC017). Thanks to Professors Wenbiao Shen and Yanjie Xie (College of Life Sciences, Nanjing Agricultural University, China) for providing the seeds of *Arabidopsis hy1-100* mutant.

## Author contributions

Z.Z., Z.C., S.B., and X.W. designed and refined the research; Z.C., S.L., T.C., and M.H. performed research; T.Y., Y.W., and Z.S. participated in the preparation of plant materials; Z.C., S.L., and T.C. analysed data; Z.C. and Z.Z. wrote the manuscript. All authors reviewed and approved the final manuscript.

## Data availability

All relevant data in this study are provided in the article and its supplementary file.

## Conflict of interests

The authors declare no conflict of interest.

## Supplementary data

Supplementary data is available at *Horticulture Research* online.

## Supplementary Material

Web_Material_uhac269
