# Supplementary material for: Haem Oxygenase 1 is a potential target for creating etiolated/albino tea plants (Camellia sinensis) with high theanine accumulation
Source: Hortic Res. 2022 Dec 2;10(2):uhac269. doi: 10.1093/hr/uhac269 (PMC10390853; doi:10.1093/hr/uhac269)
Supplement: Web_Material_uhac269 [file web_material_uhac269.doc]

**SUPPLEMENTAL INFORMATION**



**Figure S1 Relative expression of the *CsHOs* in different tissues of tea plants.** The data were obtained from the gene expression database of Tea Plant Information Archive (TPIA) (Xia et al., 2019, http://teaplant.org/).


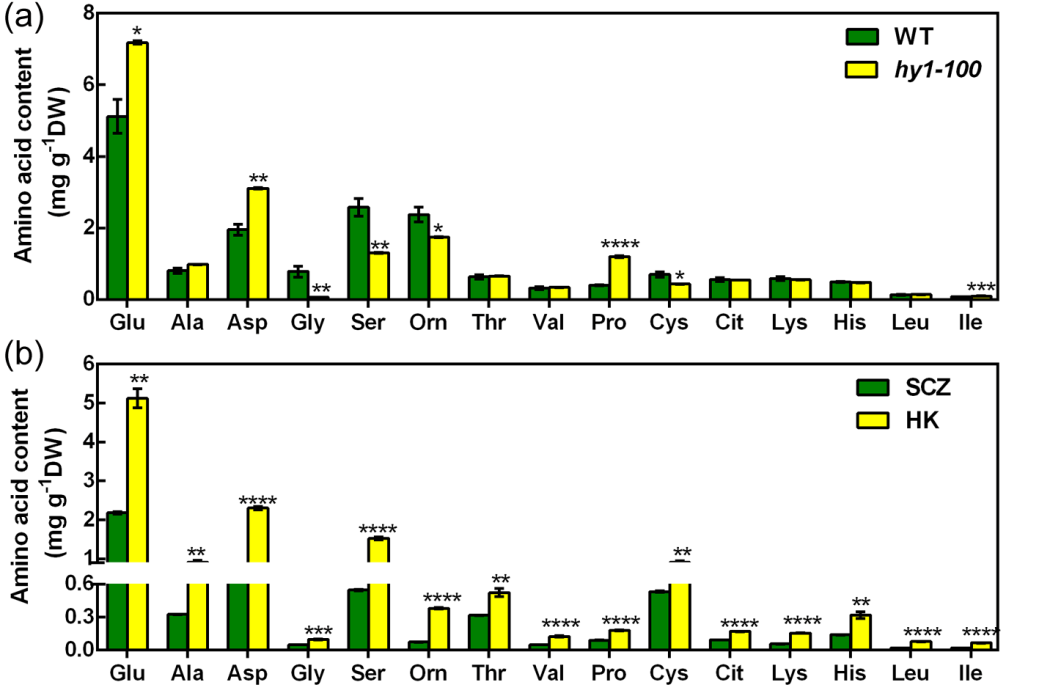
**Figure S2 Free amino acid contents in the rosette leaves of *Arabidopsis* WT and *hy1-100*, and in the leaf buds of SCZ and HK.** (a) Free amino acid contents in WT and *hy1-100* rosette leaves. (b) Free amino acid contents in the leaf buds of SCZ and HK. Glu, glutamate; Ala, alanine; Asp, aspartic acid; Gly, glycine; Ser, serine; Orn, ornithine; Thr, threonine; Val, valine; Pro, proline; Cys, cysteine; Cit, citrulline; Lys, lysine; His, histidine; Leu, leucine; Ile, isoleucine. Data are means ± SE of three biological replicates. Asterisks indicate significant differences Student’s t-test (**p* < 0.05, ***p* < 0.01, ****p* < 0.001, *****p* < 0.0001).


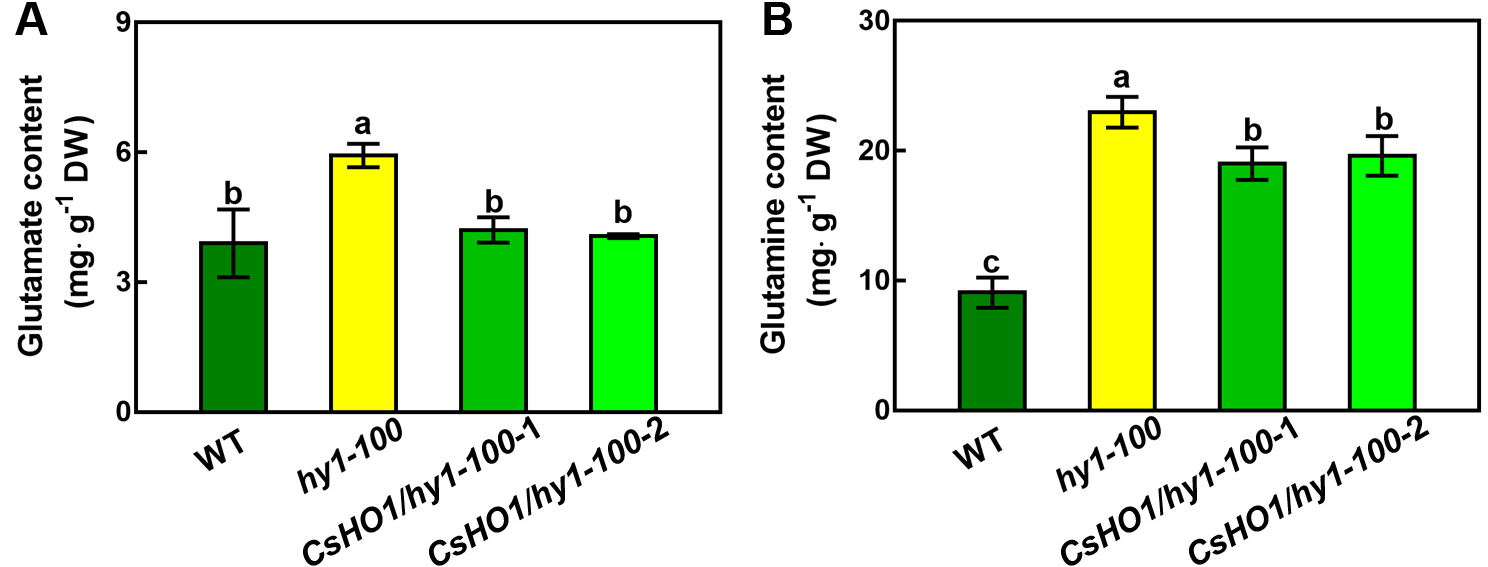


**Figure S3 The contents of glutamate and glutamine in Arabidopsis WT, *hy1-100*, and two *CsHO1* transgenic hy1-100 lines (*CsHO1*/*hy1-100*-1, *CsHO1*/*hy1-100*-2).** (A) The content of glutamate. (B) The content of glutamine.

**Table S1** The primers used in this study.

| Primer name | Locus Identify | Sequences (5’→3’) |
| --- | --- | --- |
| Xbal-CsHO1-FP |  | CCAGTCTAGAATGGCGTCAATCACACC |
| Smal-CsHO1-RP |  | ACTACCCGGGTGACAGTATTAAACGGA |
| NcoI-CsHO1-FP |  | CATGCCATGGCGTCAATCACACCCAT |
| SpeI-CsHO1-RP |  | CGGACTAGTTGACAGTATTAAACGGAG |
| Sense ODN1 |  | CGTCAATCACACCCATTTCT |
| Sense ODN2 |  | ATTGCACACCAGGGACCAGG |
| Sense ODN3 |  | GAAATTGCACACCAGGGACC |
| Sense ODN4 |  | GACAGTCTGGCAAAAGATTT |
| asODN1 |  | AGAAATGGGTGTGATTGACG |
| asODN2 |  | CCTGGTCCCTGGTGTGCAAT |
| asODN3 |  | GGTCCCTGGTGTGCAATTTC |
| asODN4 |  | AAATCTTTTGCCAGACTGTC |
| *CsGAPDH-FP* | TEA025584 | TTGGCATCGTTGAGGGTCT |
| *CsGAPDH-RP* |  | CAGTGGGAACACGGAAAGC |
| *AtACTIN7-FP* | AT5G09810 | TGGTTGGTATGGGTCAGA |
| *AtACTIN7-RP* |  | GCTTTAGGGTTAAGAGGTG |
| *CsHO1-FP* | TEA004965 | CCATTTCTCAATCCCAACC |
| *CsHO1-RP* |  | GACACCATCACCACCATCT |
